# Incidental Visceral Peritoneal and Hepatic Sarcoidosis During Routine Laparoscopic Appendectomy: A Case Report and Implications for Laparoscopic Entry

**DOI:** 10.7759/cureus.103171

**Published:** 2026-02-07

**Authors:** Sheng Dong, Matthew Seebald, Malcolm Meredith, Ruth N Reed, Fatima Khambaty

**Affiliations:** 1 Surgery, The George Washington University, Washington, D.C., USA; 2 Surgery, The George Washington University School of Medicine and Health Sciences, Washington, D.C., USA; 3 Surgery, Veterans Affairs Medical Center, Washington, D.C., USA

**Keywords:** acute appendicitis, diagnostic laparoscopy, hepatic sarcoid, intraabdominal sarcoid, laparoscopic appendectomy, peritoneal sarcoid, uncomplicated acute appendicitis

## Abstract

A 50-year-old male with a history of pulmonary sarcoidosis presented to the DC Veterans Affairs Medical Center with acute appendicitis. Laparoscopic view of the liver and peritoneum revealed studding lesions with biopsy confirmation of non-necrotizing granulomas consistent with intra-abdominal sarcoidosis. Operative and postoperative hospital courses were uncomplicated. In our case, avoidance of peritoneal sarcoid disease segments during laparoscopic entry into the abdomen may have helped reduce the chances of postoperative complications such as surgical site infection, wound dehiscence, or development of ventral hernias. However, further research into laparoscopic entry in peritoneal sarcoid patients is required to further elucidate the subject and to help guide management. Literature reviews on intra-abdominal and peritoneal sarcoids, as well as surgical implications for laparoscopic entry, were discussed.

## Introduction

Sarcoidosis is an idiopathic systemic disease that most often affects the lungs and lymph nodes, though any organ can be involved. Intra-abdominal visceral sarcoidosis manifestations have been described in the literature with varying degrees of disease burden; however, few cases of peritoneal sarcoidosis were discovered during a laparoscopic procedure [1-5]. We describe a case of incidentally found peritoneal and hepatic sarcoidosis during laparoscopic appendectomy for acute appendicitis. Laparoscopic visualization and description of these intra-abdominal sarcoidosis lesions help provide valuable insights into how sarcoidosis can present in patients. Literature reviews on intra-abdominal and peritoneal sarcoid, as well as on surgical implications for laparoscopic entry, were discussed.

## Case presentation

A 50-year-old male with a history of pulmonary sarcoidosis, GERD, constipation, sleep apnea, and migraines presented to the Washington DC Veterans Affairs (VA) Medical Center with complaints of five days of diffuse abdominal pain that was concentrated in the periumbilical and right lower quadrant of his abdomen. He experienced associated nausea and vomiting for the past one day. She denied any fevers, chills, chest pain, shortness of breath, or changes to bowel/bladder habits.

**Figure 1:**
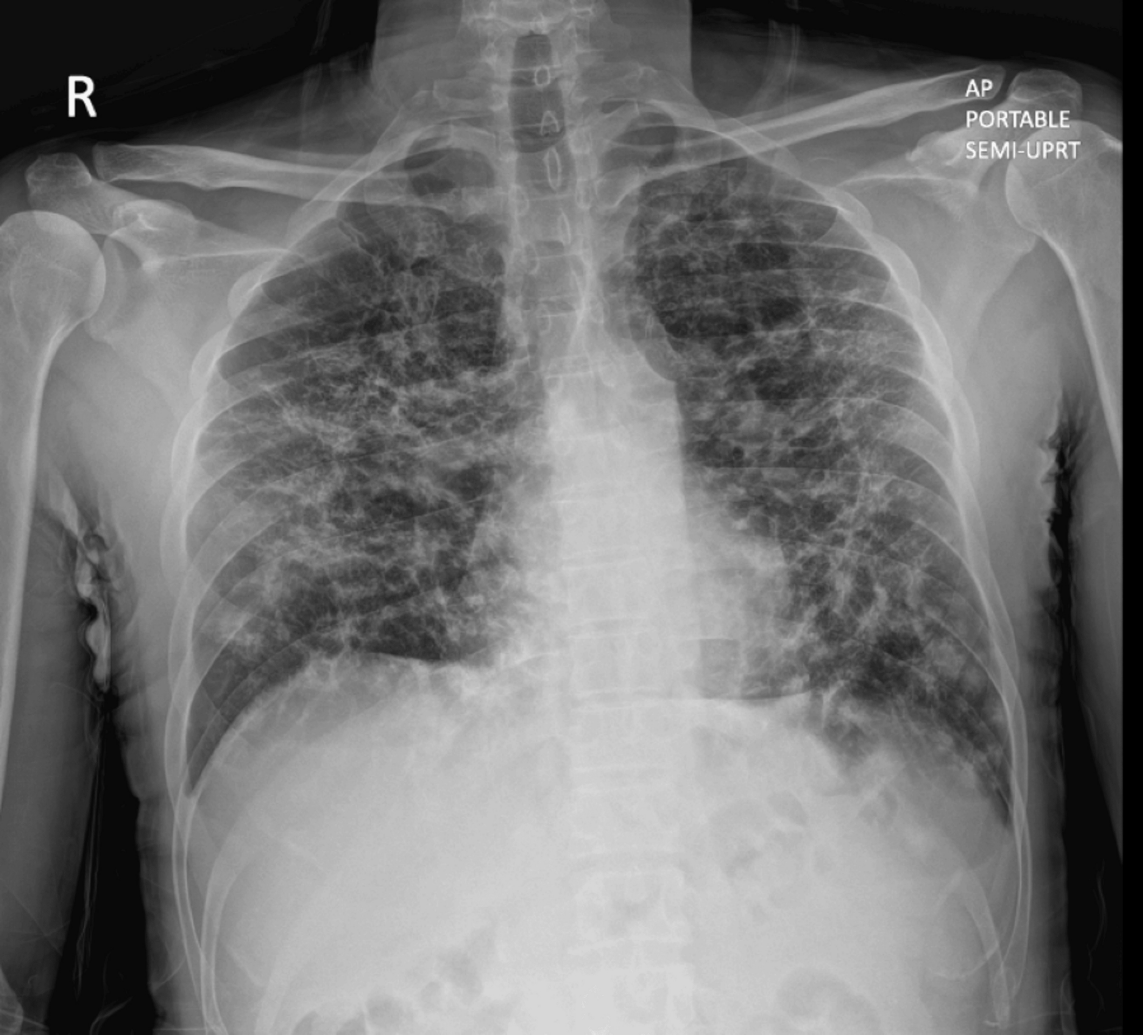
Chest X-Ray demonstrating a reticular interstitial pattern with symmetric reticulonodular opacities suggestive of advanced fibrosis and architectural distortion from sarcoidosis

On arrival, the patient was hemodynamically stable; examination revealed a soft, nondistended abdomen with right lower quadrant tenderness. Vital signs and laboratory results on arrival are detailed in Table 1 and Figure 1.

**Table 1:** Vitals and laboratory results on presentation to the VA emergency department BMI: Body Mass Index; WBC: White Blood Cell

Vitals	Value	Reference Range
Tmax (°F)	98.4	97.8–99.1
Heart rate (beats per minute)	60–80 s	60–100
Respiratory rate (breath per minute)	12	12–18
Blood pressure (mmHg)	102/70	90–120/60–80
O_2_ saturation (% O_2_)	100% room air	99–100%
BMI (kg/m^2^)	22	18.5–30
Laboratory Values		
WBC (×10^3^/µL)	9.1	4.0–11.0
Hemoglobin (g/dL)	15.3	11.7–15.5
Platelets (×10^9^/L)	192	165–415
Sodium (mmol/L)	136	135–145
Potassium (mmol/L)	4.5	3.5–5.0
Chloride (mmol/L)	99	95–105
Bicarbonate (mmol/L)	24	23–30
Blood urea nitrogen (mg/dL)	11	10–20
Creatinine (µmol/L)	1.21	0.7–1.3

The patient's sarcoidosis was diagnosed in 2014 via bronchoscopic biopsy and involved both pulmonary and cutaneous manifestations. He followed with pulmonology extensively for management of his outpatient immunosuppressive regimens, at one point requiring daily regimens of 60 mg prednisone orally in 2020, later transitioning to only methotrexate 10 mg, which did not address his symptoms of dry cough, shortness of breath, and fatigue, ultimately requiring him to resume the prednisone. Starting in 2021, the patient was then trialed on azathioprine 150 mg, which led to better symptomatic management and improvements in his pulmonary function tests, with FVC increasing by 13% and FEV1 by 10% within a year. During this time, he maintained a vigorously active lifestyle (BMI 22, 20k steps per day, walking pace, one hour of treadmill per day) but still struggled with exertional dyspnea whenever he needed to run or climb steps. He had no smoking history, no alcohol or illicit drug use, maintained a low-stress office job, and denied any exposure to toxic or hazardous chemicals during his time in the military.

**Figure 2:**
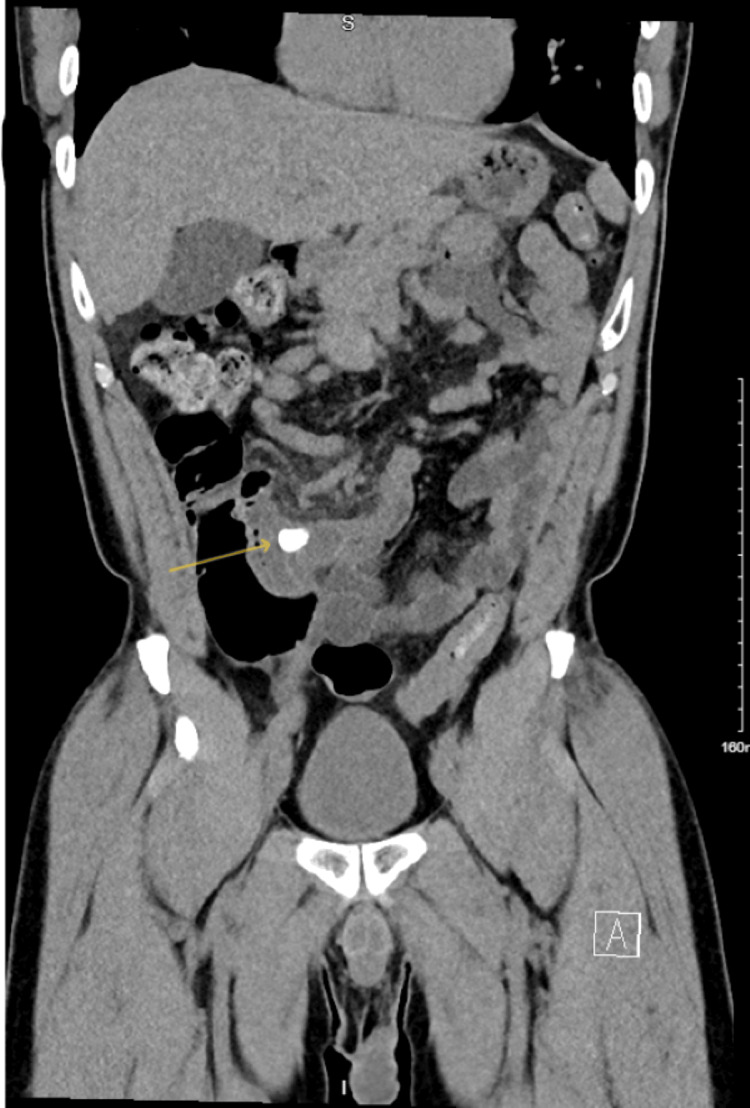
CT abdomen pelvis with contrast shows a dilated appendix of 1.6 cm with a 14 mm appendicolith (yellow arrow) and localized inflammatory stranding suggestive of acute appendicitis

A CT of the abdomen and pelvis with IV contrast demonstrated a tubular, fluid-filled structure in the right lower quadrant measuring up to 16 mm, containing a 14 mm calcification and surrounded by inflammatory stranding, consistent with acute appendicitis with appendicolith (Figure 2). Multiple bilateral pulmonary nodules up to 2 cm, with interlobular septal thickening, were also noted, consistent with his known sarcoidosis and prior imaging. The readings of the CT scan did not note any peritoneal or intra-abdominal masses, nor did they report any inflammatory changes to the liver or peritoneum.

**Figure 3:**
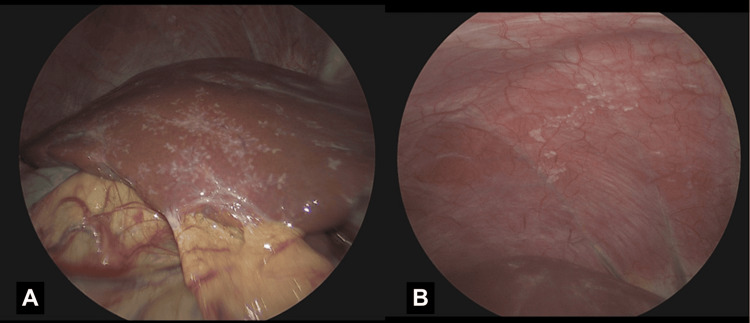
Laparoscopic view demonstrating (A) a diffuse studding pattern of the anterior liver and (B) Glisson's capsule of the visceral peritoneum anterior to the liver

The patient was started on intravenous antibiotics and sent for a laparoscopic appendectomy the next morning. The anesthesia and intubation course was uncomplicated. Intraoperatively, numerous small (<2 mm) white lesions were visualized on the liver surface and a 2 × 2 cm area of diaphragmatic peritoneum immediate upon entry and inspection of the abdomen (Figures 3A, 3B). Firstly, we directed our attention to the appendix. The appendix lay anterior to the cecum with a surrounding inflammatory rind that was dissected off of the cecal base, and the fecalith was identified. The fecalith was then milked back more distally into the appendix to clear the appendiceal base for a staple fire. A 75 mm blue load stapler was fired across the base, and another blue load was used to take the mesoappendix and associated vasculature. The lower right quadrant of the abdomen was irrigated, staple lines were reinspected, and hemostasis was achieved. Attention was then turned towards the liver and the peritoneal studding. Samples were taken using a Maryland grasper and electrocautery. Biopsies of the hepatic capsule and diaphragmatic peritoneum were submitted for permanent sections. A thorough inspection of the rest of the peritoneum and abdomen was performed, and no other evidence of studding was found. The postoperative course was unremarkable, as the patient recovered well and was discharged home later that day.

Appendiceal biopsy results revealed acute-on-chronic appendicitis with periappendicitis. The biopsy results of both the hepatic tissue and peritoneal lesions revealed small, non-necrotizing epithelioid granulomas consistent with the patient's history of sarcoidosis. PAS (periodic acid-Schiff), GMS (Gomori methenamine silver), and AFB (acid-fast bacilli) stains were all negative.

## Discussion

We report a case of hepatic and peritoneal sarcoidosis in a 50-year-old male patient during an episode of acute appendicitis. Medical management with corticosteroids remains the mainstay of treatment. Though the patient did not take steroids at the time of his surgery, he was asymptomatic from his sarcoidosis and had thorough follow-up with his pulmonologist and primary care physicians for medication management. Laparoscopy demonstrated stud-like appearances to the liver that seemed to translocate to the peritoneum in contact with the anterior liver edge. There was no other gross evidence of sarcoidosis elsewhere on the peritoneum or on any other intra-abdominal organs during the case.

Sarcoidosis is a multisystem disorder characterized by systemic noncaseating granulomas. It most often manifests in the lungs and lymph nodes, but abdominal involvement is present in only approximately 5%-15% of extrapulmonary cases [1,2]. Peritoneal sarcoidosis has been documented in only a few case reports and is rarely found in isolation, as it almost always reflects broader systemic granulomatous activity in other organ systems. Patients are more commonly women aged 20-40 and present with symptoms of ascites and abdominal pain [3-5].

The exact pathophysiology of sarcoidosis remains undetermined, but it is thought to be a dysregulated immune response to various environmental antigens in genetically susceptible patients. Extrapulmonary spread is thought to follow hematogenous and lymphatic tracts, as histopathological evidence has shown granuloma formation along bronchovascular bundles into lymphatic and hematogenous channels [6].

Peritoneal sarcoidosis can mimic peritoneal carcinomatosis and tuberculous peritonitis, which necessitates a biopsy-proven diagnosis. In this case, biopsy specimens revealed well-formed, noncaseating granulomas consistent with sarcoidosis. Furthermore, the patient had a well-documented history of treatment-refractory sarcoidosis, a prior negative carcinomatosis workup including routine labs, imaging, and colonoscopies, and no history of carcinogenic exposure, travel, or systemic signs of infection outside of this episode of appendicitis.

Surgical implications for patients with peritoneal sarcoidosis in laparoscopic or open surgical approaches to entering the abdomen remain unclear. In theory, granulomatous inflammation in sarcoidosis, including involvement of extrapulmonary organs, can impair normal wound healing due to chronic immune activation and tissue remodeling [7,8]. However, there has been little to no literature to date documenting the incidence or disease burden of incisional hernias, surgical site infections, or wound dehiscence in patients with peritoneal sarcoidosis.

Locality of the peritoneal sarcoid disease, patient compliance with immunosuppressive medication regimens, and overall disease burden may impact laparoscopic entry decisions regarding postoperative wound complications. In our case, the patient's focal right upper quadrant peritoneal studding was unlikely to alter our laparoscopic approach for an appendectomy, as we were able to introduce a Veress needle through a supraumbilical incision to introduce a 10 mm port with two smaller 5 mm ports in the suprapubic abdomen and right lower abdomen, well far away from the peritoneal sarcoid burden. Outpatient clinic follow-up with this patient also did not demonstrate any evidence of complications from the procedure. However, several cases of peritoneal sarcoidosis have been reported, with a more significant peritoneal disease burden, including diffuse nodular thickening and omental caking. These patients did not require laparoscopic surgery at the time of diagnosis; therefore, there were no reports of postsurgical complications [1,9]. Overall, there is a lack of literature and of large-scale, controlled studies directly comparing abdominal surgical complication rates in patients with sarcoidosis versus controls, and further research is needed to clarify these risks.

## Conclusions

We report a case of hepatic and peritoneal sarcoidosis in a 50-year-old male during an episode of acute appendicitis. Sarcoidosis can have extrapulmonary systemic dissemination of granulomatous inflammation to the abdomen in approximately 10% of cases. A tissue biopsy is required to rule out mimickers such as peritoneal carcinomatosis or tuberculosis. While patients remain asymptomatic, peritoneal sarcoidosis remains a rare clinical manifestation with possible surgical implications for wound healing under chronic inflammatory changes and tissue remodeling. In patients with localized peritoneal sarcoidosis, avoidance of diseased segments during laparoscopic entry may anecdotally reduce chances of wound complications; however, further higher-level evidence is required to guide definitive management. Further studies into surgical site outcomes in sarcoidosis patients with disease burden to the peritoneum undergoing abdominal surgery are required to elucidate postoperative management recommendations.
